# A Qualitative Analysis of Contributory Factors to Serious Incidents Involving Adults With Learning Disabilities Receiving NHS Mental Healthcare

**DOI:** 10.1192/bjo.2024.185

**Published:** 2024-08-01

**Authors:** Nusra Khodabux, Reza Kiani

**Affiliations:** Leicestershire Partnership NHS Trust, Leicester, United Kingdom

## Abstract

**Aims:**

This study aimed to analyse contributory factors to serious incidents (SIs) involving adult patients with intellectual disabilities receiving NHS care in a mental health trust. People with intellectual disabilities face considerable preventable harm and disparities in care. In-depth analysis of contributory factors to incidents involving adults with an intellectual disability, using human-factors based frameworks is lacking. Individual SI reports contain useful data, but learning is often limited without aggregated analysis.

**Methods:**

Thirty anonymized serious incident reports (2014–2023) from an NHS mental health Trust's intellectual disability service were analysed qualitatively using the Yorkshire Contributory Factors Framework, followed by reflexive thematic analysis (RTA) to identify patterns across the data. This enabled nuanced themes to emerge across errors at the sharp end and systems-level factors at the blunt-end.

**Results:**

Across 30 reports, 606 discrete factors were identified. Situational factors such as behavioural escalations and staff competency gaps were most frequent (n = 187, 31%). Other factors included active failures, such as slips, lapses, mistakes, violations (n = 109, 18%), organisational influences (n = 107, 18%), communication breakdowns (n = 75, 12%), unfavourable working conditions (n = 62, 10%), cultural factors such as reluctance to voice safety concerns (n = 51, 8%), and external system factors (n = 15, 2%).

Using RTA, we identified recurring themes across incidents involving interactions between sharp-end human and blunt-end system factors, with broader issues shaping frontline performance. Patient marginalisation, excessive workloads, lack of resources, and cultures tolerant of shortcuts aligned to permit errors. Deficient coordination across fragmented healthcare systems and overdependence on non-permanent workers and bank staff obstructed comprehensive incident reviews. Failure to adequately probe cultural influences and external pressures further reflect the limited extent of investigational efforts.

**Conclusion:**

Adults with intellectual disabilities are subject to serious incidents caused by interacting human and system-level factors, including organisational, cultural and external factors. Addressing under-resourcing and improving investigation quality are paramount to enhancing safety of care for people with intellectual disabilities.

